# Genetic Markers of Obesity Risk: Stronger Associations with Body Composition in Overweight Compared to Normal-Weight Children

**DOI:** 10.1371/journal.pone.0019057

**Published:** 2011-04-15

**Authors:** Andreas Beyerlein, Rüdiger von Kries, Andrew R. Ness, Ken K. Ong

**Affiliations:** 1 Institute of Social Paediatrics and Adolescent Medicine, Ludwig-Maximilians University of Munich, Munich, Germany; 2 School of Oral and Dental Science, Bristol, United Kingdom; 3 MRC Epidemiology Unit, Institute of Metabolic Science, Cambridge, United Kingdom; University of Ottawa, Canada

## Abstract

**Background:**

Genetic factors are important determinants of overweight. We examined whether there are differential effect sizes depending on children's body composition.

**Methods:**

We analysed data of n = 4,837 children recorded in the Avon Longitudinal Study of Parents and Children (ALSPAC), applying quantile regression with sex- and age-specific standard deviation scores (SDS) of body mass index (BMI) or with body fat mass index and fat-free mass index at 9 years as outcome variables and an “obesity-risk-allele score” based on eight genetic variants known to be associated with childhood BMI as the explanatory variable.

**Results:**

The quantile regression coefficients increased with increasing child's BMI-SDS and fat mass index percentiles, indicating larger effects of the genetic factors at higher percentiles. While the associations with BMI-SDS were of similar size in medium and high BMI quantiles (40th percentile and above), effect sizes with fat mass index increased over the whole fat mass index distribution. For example, the fat mass index of a normal-weight (50th percentile) child was increased by 0.13 kg/m^2^ (95% confidence interval (CI): 0.09, 0.16) per additional allele, compared to 0.24 kg/m^2^ per allele (95% CI: 0.15, 0.32) in children at the 90th percentile. The genetic associations with fat-free mass index were weaker and the quantile regression effects less pronounced than those on fat mass index.

**Conclusions:**

Genetic risk factors for childhood overweight appear to have greater effects on fatter children. Interaction of known genetic factors with environmental or unknown genetic factors might provide a potential explanation of these findings.

## Introduction

Increasing prevalence of childhood overweight has been reported worldwide [1]. Genetic factors are important determinants of the overweight risk as has been shown in adoption and twin studies [2], [3] and in observational studies pointing to the important role of maternal body mass index (BMI) in the development of overweight in children [4], [5].

Recent genome-wide association (GWA) studies allowed identifying several genetic factors associated with childhood and adult obesity, such as variants of the FTO and MC4R genes [6], [7]. Members of our study group recently combined eight genetic variants (which had shown individual associations with childhood BMI in previous studies) to a so-called “obesity-risk-allele score” and found strong statistical evidence for positive associations of this score with mean BMI and body fat mass at the age of 9 years [8].

Similarly, shifts in mean BMI have been observed for environmental factors which, upon more detailed scrutiny, were mainly caused by shifts in the upper tail of the distribution [9], [10]. For example, we found that, while the middle part of the BMI distribution was similar at the age of 5–6 years in formerly breastfed and formula fed children, the lower tail showed higher values in breastfed children, and the upper tail lower values [11]. These analyses were performed with the use of quantile regression [12], [13], a statistical method that offers a more comprehensive approach than the widely used linear regression. While linear regression focuses on shifts of the mean, quantile regression allows differentiating shifts in different parts of the distribution.

We therefore hypothesized that effect sizes of genetic risk factors for overweight might be stronger in children with high compared to children with normal or low BMI or fat mass. In order to answer this question, we assessed BMI and fat mass dependent associations of genetic risk factors for childhood obesity by quantile regression.

## Materials and Methods

### Data

The Avon Longitudinal Study of Parents and Children (ALSPAC) is a longitudinal birth cohort study of the determinants of development, health, and disease during childhood and beyond and has been described in more detail elsewhere [14]. Initially, 14,541 pregnant women with an expected date of delivery between April 1991 and December 1992 were enrolled; 13,971 of their children formed the original cohort at 1 y of age. Detailed information has been collected using self-administered questionnaires, data extraction from medical notes, and linkage to routine information systems and at research clinics. Ethical approval for the study was obtained from the ALSPAC Law and Ethics Committee and Local Research Ethics Committees. Publication of the final paper has been approved by the ALSPAC board. The Ethics Committee of the Physicians' Chamber of Bavaria waived the need for consent, since this study was based on analyses of anonymized data.

Childhood weight and height was measured annually between ages 7 and 11 y at dedicated ALSPAC Focus clinics by a trained research team. Height was measured to the nearest 0.1 cm using a Leicester Height Measure (Holtain Crosswell, Dyfed) and weight while wearing underwear was measured to the nearest 0.1 kg using Tanita electronic scales. Fat mass and fat-free mass was assessed (only) at the 9-year-old research clinic visit (at which 7,725 of the children were seen) by whole body dual energy X-ray absorptiometry (DXA) (Prodigy scanner, Lunar Radiation Corp, Madison, Wisconsin, US).

We calculated BMI as weight/height^2^ (kg/m^2^). To adjust for sex and age, we transformed the observed BMI values to sex- and age-specific standard deviation scores (SDS) established by the World Health Organisation (WHO, available at: http://www.who.int/growthref/en/) using the LMS method [15]. The position of children's BMI values within the distribution (the quantile) did not change considerably by the age- and sex-adjusted transformation to BMI-SDS. For descriptive analyses, we defined overweight and obesity according to BMI reference values of the *International Obesity Task Force (IOTF)* [16]. We calculated fat and fat-free mass indices for each child from DXA measurements at age 9 y by dividing fat mass and fat-free mass (kg) by height squared (m^2^) [17].

Genotype information was available for 7,333 children with respect to six GWA-obesity variants previously reported to show association with BMI or obesity in children [6], [7], [18]; these variants were: rs9939609 (in/near to FTO); rs17782313 (MC4R), rs6548238 (TMEM18), rs10938397 (GNPDA2), rs368794 (KCTD15), rs2568958 (NEGR1). We further included the variants rs925946 (BDNF) and rs7647305 (ETV5) in our analyses which had been reported to be associated with BMI in adults [19] and were found to be associated with overweight in children in our previous study [8]. As in the latter study, we calculated an “obesity-risk-allele score” by counting the total number of obesity risk alleles across these eight genetic variants. Only one variant at each locus was chosen and only individuals with complete genotype data at all eight variants were included in the obesity-risk-allele score analyses. This score approximated a normal distribution and showed a linear association with BMI SDS at age 9 y [8]. We did not make use of a “weighted obesity-risk-allele score” (with weighted contributions of each variant according to their apparent effect size on adult BMI), since such a weighted score showed essentially the same associations as the unweighted score in our previous study [8].

We restricted our analyses to singleton white Europeans plus one randomly selected child from each mother for whom more than one child had entered the study (n = 7,146 children). In total, the dataset contained n = 4,837 observations with full information on both BMI at 9 years and the obesity-risk-allele score, n = 4,613 of which had also measurements of fat mass and fat-free mass recorded.

### Statistical analyses

Quantile regression is a statistical approach of modelling different sample percentiles (‘quantiles’) of an outcome variable by a number of explanatory variables [12], [20], [21]. The results of quantile regression are interpreted in a similar way to those of linear regression. While linear regression models the mean of the outcome distribution, quantile regression models selected quantiles, e.g. the 90^th^ percentile (0.90 quantile) - and, like linear regression, uses all available data, irrespective of the percentile modelled. In both cases, regression coefficients quantify potential effects on the specific parameter (mean or quantile) of the outcome distribution on a population level. This means that linear regression coefficients for a binary risk factor can be interpreted as difference of the mean value of the outcome distribution between subjects exposed and not exposed. Similarly, quantile regression coefficients for a binary risk factor represent the difference of the respective quantile in the estimated outcome distribution in subjects exposed vs. not exposed (irrespectively of how many exposed and not exposed subjects lie above or below the respective quantile). In summary, quantile regression leads to more comprehensive results, because it allows to assess any part of the outcome distribution.

We calculated separate quantile regression models with either BMI-SDS, fat mass index or fat-free mass index as outcome variable and the obesity-risk-allele score as explanatory variable, assessing the 0.03, 0.1, 0.2,…, 0.9, and 0.97 quantiles of the respective outcome variable. We adjusted models with fat mass index and fat-free mass index as an outcome for sex, age and height. Models for BMI-SDS were initially not adjusted for sex and age, since BMI-SDS was defined based on sex- and age-specific transformations. In a sensitivity analysis, we examined whether adjustment for sex and age changed the results from the models for BMI-SDS.

We calculated 95% confidence intervals (CIs) for quantile regression effect estimates using bootstrap methods [12], [20]. Additionally, we compared the quantile regression results with those from linear regression models. This approach has been used in other quantile regression related literature [9], [12], [20]. To enable direct comparison to ordinary linear regression, we did not examine non-linear effects (such as polynomial splines) in the quantile regression models.

All calculations were carried out with the statistical software R 2.6.2 (http://cran.r-project.org), using the *quantreg* package.

## Results

The children analysed had a mean BMI of 17.6 kg/m^2^, a mean BMI-SDS of 0.35 and a mean fat mass index of 4.3 kg/m^2^ at 9 years of age (table 1). The prevalence of overweight (including obesity) and obesity according to IOTF criteria was 20.5% and 3.9%, respectively. Children excluded due to missing genotype data were similar with respect to mean values of BMI (17.8 kg/m^2^), BMI-SDS (0.40) and fat mass index (4.4 kg/m^2^).

**Table 1:** Characteristics of the study population (n = 4,837).

Variable	Mean (SD)/n (%)
Children's BMI [kg/m^2^]	17.6 (2.8)
Children's BMI standard deviation score (SDS)	0.35 (1.14)
Fat mass index [kg/m^2^] *	4.3 (2.4)
Fat-free mass index [kg/m^2^] *	12.6 (1.0)
Age [y]	9.9 (0.3)
Girls	n = 2,450 (50.7%)
Overweight/obese children **	n = 993 (20.5%)
Obese children **	n = 191 (3.9%)

*n = 4,613

**classified using IOTF cut-off values [16]

The linear regression estimates indicated a mean increase of 0.08 units (95% confidence interval (CI): 0.07, 0.10) in BMI-SDS and of 0.13 kg/m^2^ (95% CI: 0.09, 0.16) in fat mass index per allele increase in the obesity-risk-allele score (table 2). These values were almost identical to those obtained by median (50^th^ percentile) regression. The respective quantile regression coefficients were positive for any BMI-SDS or fat mass index percentile. This indicates that the obesity-risk-allele score was associated with shifts to higher values in any category of both BMI-SDS and fat mass index.

**Table 2:** Regression coefficients [95% confidence intervals] of the obesity-risk-allele score on sex- and age-specific BMI-SDS or fat mass index [kg/m^2^] at 9 years as estimated by linear regression (LR) and quantile regression at specific percentiles p.

Outcome variable	LR	0.03p	0.10p	0.20p	0.30p	0.40p	0.50p	0.60p	0.70p	0.80p	0.90p	0.97p
BMI-SDS	0.08	0.04	0.06	0.06	0.07	0.09	0.10	0.10	0.10	0.10	0.09	0.09
	[0.07, 0.10]	[0.00, 0.08]*	[0.03, 0.08]	[0.04, 0.08]	[0.05, 0.10]	[0.07, 0.11]	[0.07, 0.12]	[0.08, 0.12]	[0.07, 0.13]	[0.06, 0.13]	[0.06, 0.13]	[0.05, 0.13]
Fat mass index	0.13	0.04	0.04	0.05	0.07	0.10	0.13	0.17	0.17	0.19	0.24	0.38
	[0.09, 0.16]	[0.01, 0.06]	[0.02, 0.06]	[0.03, 0.07]	[0.05, 0.10]	[0.07, 0.13]	[0.10, 0.17]	[0.12, 0.21]	[0.11, 0.22]	[0.12, 0.25]	[0.15, 0.32]	[0.26, 0.49]
Fat-free mass index	0.03	0.00	0.01	0.03	0.03	0.02	0.02	0.03	0.04	0.03	0.03	0.07
	[0.01, 0.04]	[-0.03, 0.02]	[-0.01, 0.03]	[0.01, 0.05]	[0.01, 0.04]	[0.01, 0.04]	[0.01, 0.04]	[0.01, 0.05]	[0.02, 0.06]	[0.01, 0.05]	[0.01, 0.06]	[0.03, 0.12]

*p>0.05

Models with fat mass index and fat-free mass index as outcome variable were adjusted for sex, age and height.

**Figure 1:**
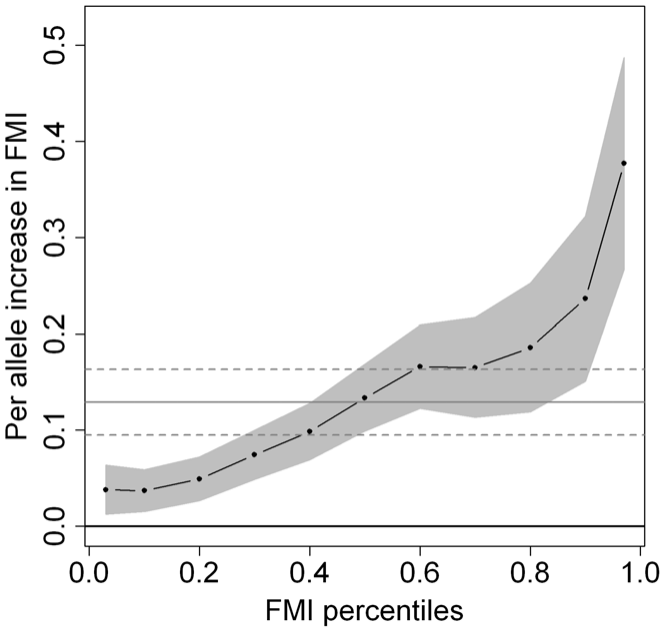
Point estimates and 95% confidence bounds (grey areas) for increase in fat mass index (FMI) at 9 years per obesity-risk-allele (n = 4,613). The dots represent specific FMI percentiles (0.03 percentile, 0.1 to 0.9 deciles, and 0.97 percentile) in the quantile regression model with adjustment for sex, age and height and are connected by dashes to visualize trends by outcome percentiles. The grey horizontal lines represent the linear regression coefficients and their respective confidence intervals (dashed).

However, the estimated effects increased by child's BMI-SDS and fat mass index percentiles, pointing to an additional shift of the upper percentiles. While the associations were of similar size in medium and high BMI-SDS quantiles (40^th^ percentile and above), increasing effect sizes over the whole distribution were observed with respect to children's fat mass index (figure 1). For example, the fat mass index of a normal-weight (50^th^ percentile) child was increased by 0.13 kg/m^2^ (95% CI: 0.09, 0.16) per additional allele of the obesity-risk-allele score. This risk factor was associated with an average difference of 0.24 (95% CI: 0.15, 0.32) kg/m^2^ in children at the 90^th^ percentile and of 0.38 (95% CI: 0.26, 0.49) units at the 97^th^ percentile per allele. The effects on fat-free mass index were weaker compared to those on fat mass index, and there was no clear pattern of increasing effect sizes by fat-free mass index percentiles. The sensitivity analyses for BMI-SDS with adjustment for sex and age yielded virtually identical results compared to those without adjustment (data not shown).

## Discussion

Our analyses showed evidence of weight status dependent effects of genetic risk factors for overweight on body composition: In general, the obesity-risk-allele score was associated with an increase in any part of the BMI and fat mass distributions. However, particularly with respect to body fat mass, the effect size was directly modified by the percentile of the outcome variable. These results suggest that genetic risk factors influence body composition not only continuously over the whole distribution, but also to a stronger extent in heavier children. An additional implication of our findings might lie in risk prediction. If gene-environment, and gene-gene, interactions have such marked effects on fat mass as suggested by our findings, it is therefore possible that genetic variants and environmental determinants might have far stronger predictive abilities for obesity among children who are already overweight, or those with obese parents.

The mechanisms by which certain genetic variants contribute to higher body mass are still largely unclear. We hypothesize that our findings reflect one of the following two potential mechanisms: Firstly, hitherto unknown obesity risk genes could interact with, or modify, the effects of the genetic factors examined in this study. However, as yet there is little evidence for marked gene-gene interactions between known disease-related variants. Interestingly, one study reported differential associations of the FTO gene for Whites and African-Americans with respect to obesity [22]. Although this finding cannot help to explain our results, since we restricted our analyses to white children, it points to potential interactions of genetic factors with respect to obesity. Unfortunately, we are not aware of other studies of this kind in the context of obesity. Advanced statistical approaches such as random forests may be helpful in identifying interacting genes in GWA studies, as demonstrated with respect to rheumatoid arthritis susceptibility [23].

Another potential explanation is that genetic risk factors for overweight may cause an increased susceptibility to certain environmental obesity risk factors. The presence of gene-environment interactions could also explain the similar quantile regression patterns found for environmental risk factors of overweight in previous studies [9], [10]. Specifically, it has been shown that overweight children with a specific MC4R variant were not able to maintain their weight loss achieved during a lifestyle intervention in contrast to children without these mutations [24]. Further studies of this kind might provide further evidence for gene-environment interactions.

A particular strength of our study is the appliance of quantile regression which offers a more comprehensive approach than linear regression. While linear regression focuses on shifts of the mean which may be caused by a true shift of the mean with a shift of the entire distribution or a shift in the upper tail or lower tail only, quantile regression allows differentiating shifts in different parts of the distribution. Therefore, this approach enabled us to reveal an additional shift of the upper percentiles of BMI-SDS and fat mass in children, additionally to the previous shown mean shift of these two outcomes [8].

A potential limitation of our study might consist in the high drop-out rate (44.7%) between enrolment and 9-year visit. Reasons for loss to follow-up were that children were no longer eligible, did not respond to the invitation letter for the 9-year visit, refused or failed to attend the visit. However, it is difficult to imagine why nonparticipation might account for different effects of genotype data on different parts of the body composition distribution.

Selection bias due to children with missing genotype data should not be a major issue with respect to our analyses, since there were no substantial differences in BMI or fat mass index at 9 years between responders and non-responders.

Potential intercorrelation in close family members is another important issue in studies assessing effects of genetic predispositions. We had therefore restricted our analyses to one child per mother in the dataset.

In conclusion, for genetic risk factors of childhood overweight, stronger associations in children with higher levels of BMI and fat mass were observed. Interaction between genetic and environmental risk factors might provide a potential explanation of these findings.
